# Effect of CRISPR/Cas9-Mediated PD-1-Disrupted Primary Human Third-Generation CAR-T Cells Targeting EGFRvIII on In Vitro Human Glioblastoma Cell Growth

**DOI:** 10.3390/cells9040998

**Published:** 2020-04-16

**Authors:** Tsutomu Nakazawa, Atsushi Natsume, Fumihiko Nishimura, Takayuki Morimoto, Ryosuke Matsuda, Mitsutoshi Nakamura, Shuichi Yamada, Ichiro Nakagawa, Yasushi Motoyama, Young-Soo Park, Takahiro Tsujimura, Toshihiko Wakabayashi, Hiroyuki Nakase

**Affiliations:** 1 Department of Neurosurgery, Nara Medical University, Kashihara 634-8521, Japan; fnishi@naramed-u.ac.jp (F.N.); K163403@naramed-u.ac.jp (T.M.); rmatsuda@naramed-u.ac.jp (R.M.); mnaka@grandsoul.co.jp (M.N.); syamada@naramed-u.ac.jp (S.Y.); nakagawa@naramed-u.ac.jp (I.N.); myasushi@naramed-u.ac.jp (Y.M.); park-y-s@naramed-u.ac.jp (Y.-S.P.); nakasehi@naramed-u.ac.jp (H.N.); 2 Grandsoul Research Institute for Immunology, Inc., Uda 633-2221, Japan; 3 Department of Neurosurgery, Nagoya University Graduate School of Medicine, Nagoya 464-8601, Japan; anatsume@med.nagoya-u.ac.jp (A.N.); wakabat@med.nagoya-u.ac.jp (T.W.); 4 Clinic Grandsoul Nara, Uda 633-2221, Japan; takahiro@grandsoul.co.jp

**Keywords:** CAR, CRISPR/Cas9, EGFRvIII, PD-1, glioblastoma

## Abstract

Glioblastoma (GBM), which is the most common malignant brain tumor, is resistant to standard treatments. Immunotherapy might be a promising alternative for the treatment of this cancer. Chimeric antigen receptor (CAR) is an artificially modified fusion protein that can be engineered to direct the specificity and function of T cells against tumor antigens. However, the antitumor effects of EGFRvIII-targeting CAR-T (EvCAR-T) cells in GBM are limited. The inhibitory effect is induced by the interaction between programmed cell death protein 1 (PD-1) on activated EvCAR-T cells and its ligands on GBM cells. In the present study, PD-1-disrupted EvCAR-T cells were established using the clustered regularly interspaced short palindromic repeats (CRISPR)/CRISPR-associated protein 9 (Cas9). The sgRNA/Cas9 expression vectors designed precisely disrupted the target region of PD-1 and inhibited the expression of PD-1 in EvCAR-T cells. The PD-1-disrupted EvCAR-T cells had an in vitro growth inhibitory effect on EGFRvIII-expressing GBM cells without altering the T-cell phenotype and the expression of other checkpoint receptors. In the future, the in vivo antitumor effect of this vector should be evaluated in order to determine if it could be applied clinically for improving the efficacy of EvCAR-T cell-based adoptive immunotherapy for GBM.

## 1. Introduction

Glioblastoma (GBM), which is the most common and aggressive brain tumor, is classified as a grade IV astrocytoma in the 2016 World Health Organization classification of tumors of the central nervous system [1]. This means that it is a glioma of the highest grade and the most malignant form. After surgical tumor resection to an extent that is safely feasible, the current treatment standard involves the use of a DNA-alkylating agent such as temozolomide and radiotherapy. However, this has limited effects in terms of the prolongation of survival [2]. In particular, GBM that produces unmethylated O6-methylguanine methyltransferase, a DNA repair enzyme, is more resistant to this therapy [3,4]. Further, the antiangiogenic agent bevacizumab (a humanized monoclonal antibody against vascular endothelial growth factor) resulted in median survival of less than 2 years in newly diagnosed GBM patients [5]. Survival among GBM patients remains poor despite attempts to improve the efficiency of treatments for GBM over the last three decades [6]. Thus, novel therapeutic approaches need to be investigated for the treatment of patients with GBM.

The chimeric antigen receptor (CAR) is an artificially modified fusion protein that commonly consists of a specificity-conferring extracellular antigen recognition domain of an antigen-specific monoclonal antibody (mAb) single-chain variable region (scFv) fused to a T-cell receptor (TcR), or an immunoglobulin-related signal domain, and a more intracellular costimulatory domain [7]. Unlike TcRs, CARs can elicit highly specific targeting of antigens in a manner that is independent of the major histocompatibility complex [8], which is often downregulated in gliomas [9]. CARs have undergone several modifications since the production of the first generation of CARs, which were genetically recombinant receptors that consisted of an scFv obtained from mAbs that were specific for certain tumor-associated antigens, a transmembrane domain, and a cytoplasmic signaling domain from the CD3ζ chain, which is a subunit of the TcR complex [10]. T cells expressing a CD28 signal domain-containing CAR have been shown to enhance interleukin (IL)-2 production and sustain T cell proliferation [11]; additionally, CAR can confer resistance against immune suppression mediated by transforming growth factor-β and regulatory T cells [12]. Further, the inclusion of domains derived from tumor necrosis factor receptor family members (4-1BB and OX40) in CARs has also been shown to enhance the cytotoxicity of CAR-expressing T (CAR-T) cells [13]. Thus, the third generation of CARs is comprised of two costimulatory domains, such as CD28 and 4-1BB, with a transmembrane domain and the CD3ζ chain. The adoptive transfer of CAR-T cells has been reported to be highly effective in the treatment of CD19-positive leukemia and lymphoma in the clinic [14,15]. The reports published so far provide a strong basis for the pursuit of CAR-T cell-based therapy for other types of cancers, including GBM [16]. However, the third generation of ERBB2 (HER-2/neu)-targeting CAR-T cells, which are controlled by 4-1BB, CD28 and CD3ζ, induced adverse events in a patient with colon cancer, who experienced respiratory distress within 15 min after the infusion of HER-2/neu-targeting CAR-T cells and had a dramatic pulmonary infiltrate on her chest radiograph [17]. Despite intensive medical interventions, the patient died 5 days after the infusion: the findings revealed that the transferred cells were immediately localized to the lung following infusion and were triggered to release a large amount of cytokines in response to low levels of ERBB2 on lung epithelial cells [17]. Thus, the presence of the target molecule of CARs, which is not expressed in normal tissues, is indispensable for the clinical application of the CAR-T cells.

Epidermal growth factor receptor variant III (EGFRvIII) is the most common variant of EGFR that has been detected in human tumors [18]. It is a tumor-specific, oncogenic, and immunogenic epitope [19] that has been detected in 50% of all EGFR-amplified GBMs and is rarely observed in normal tissues [20,21]. An analysis of data from GlioVis (http://gliovis.bioinfo.cnio.es/) revealed that GBM accounts for 38.3% of total gliomas. Thus, EGFRvIII-positive GBM was estimated to comprise 19.2% of total gliomas. In addition, EGFR, programmed cell death protein 1 (PD-1), and PD-L1 are detected in 66.7%, 2.5%, and 0.1% of gliomas, respectively, and the corresponding detection rates in GBM were estimated as 63.1%, 4.9%, and 0.0%, respectively (Figure S2). We previously reported about a novel lentiviral-based vector for a CAR that consists of an EGFRvIII-specific scFv (clone: 3C10) coupled to the motifs of the CD3ζ chain, the CD8 transmembrane domain, and the co-stimulatory 4-1BB and CD28 domains (pELNS-3C10-CAR) [22]. Further, EGFRvIII-targeting CAR-T (EvCAR-T) cells have been found to successfully elicit GBM regression under in vivo settings, with the effect being enhanced in the presence of lenalidomide [23]. In addition, transduction of the pELNS-3C10-CAR carried by lentivirus in the human natural killer cell line KHYG-1 was found to potentiate natural killer cell-mediated growth inhibition and apoptosis in GBM cells [24].

Omuro et al. reported that administration of nivolumab (an anti-PD-1 antibody) with or without ipilimumab (an anti-CTLA-4 antibody) in patients with recurrent GBM was safe and well-tolerated, but had limited anti-tumor effects on GBM [25]. The limited antitumor effect of the anti-PD-1 antibody in GBM might be the result of low expression of or a lack of PD-1 ligands, including PD-L1, in the GBM cells. In fact, GlioVis-based analysis revealed that only 2.5% and 4.9% of total glioma and GBM samples, respectively, were positive for PD-L1 (Figure S2). However, a clinical trial showed that PD-L1 expression could be upregulated in GBM cells by induction of GBM cell response to EvCAR-T cell infiltration in GBM. However, this response could inhibit EvCAR-T cell function and help GBM escape from the EvCAR-T cells [26]. This means that blocking the PD-1/PD-L1 pathway in the immunosuppressive GBM microenvironment with an anti-PD-1 antibody could potentially enhance and prolong the antitumor effect of EvCAR-T cells. Thus, simultaneous administration of CAR-T cells and an anti-PD-1 antibody might be a promising approach, since GBM cells are induced to express PD-L1 in response to CAR-T cells. However, Arlauckas et al. reported that although anti-PD-1 antibodies effectively bind PD-1 on CD8-positive tumor-infiltrating T cells, these antibodies are only effective early after treatment, as they are later captured from the T-cell surface by tumor-associated macrophages [26]. Thus, such a strategy could only have a transient effect. For sustaining the antitumor effect of EvCAR-T cells in PD-L1-expressing GBM, permanent inhibition of the PD-1 gene in EvCAR-T cells might be the optimal approach. 

Based on all the related findings reported so far, the establishment of PD-1-disrupted EvCAR-T cells is the basis of the present study. We produced such EvCAR-T cells with the help of a tool that uses clustered regularly interspaced short palindromic repeats (CRISPRs) and CRISPR-associated protein 9 (Cas9), which are known to disrupt the target gene via small RNAs that guide the Cas9 DNA nuclease to the target site through base pairing, as this approach has been shown to be highly specific and efficient for engineering and disrupting eukaryotic genomes [27,28]. The aims of this study are to induce PD-1 gene-disrupted EvCAR-T cells from human peripheral blood T cells with the help of a CRISPR/Cas9-based genome editing tool and to evaluate the in vitro antitumor effects of PD-1-disrupted EvCAR-T cells on human GBM cells in real time.

## 2. Materials and Methods

### 2.1. Ethics Statements

All the experimental methods were carried out in keeping with the requisite guidelines. Peripheral blood was collected from a healthy volunteer with the approval of, and in accordance with the guidelines of, the Ethics Committee of Nara Medical University (Number 1068), and informed consent was obtained according to the tenets of the Declaration of Helsinki.

### 2.2. Cell Lines

Cells of the human embryonic kidney cell line HEK293T were purchased from the American Type Culture Collection (ATCC; Manassas, VA, USA). Two GBM cell lines were used in this study: U-251MG is a cell line that does not express EGFRvIII [29], and it was purchased from the Japanese Collection of Research Bioresources (JCRB) cell bank (Osaka, Japan). The other one was the EGFRvIII-expressing DKMG (Ev-DKMG) cell line, which was purchased from Celther POLSKA (Lodz, Poland). Cell lines were maintained in Dulbecco’s modified Eagle medium (DMEM; Thermo Fisher Scientific Inc., Waltham, MA, USA) supplemented with 10% heat-inactivated fetal bovine serum (FBS; MP Biomedicals, Santa Ana, CA, USA), 100 units/mL of penicillin, and 100 μg/mL of streptomycin (Thermo Fisher Scientific Inc.) at 37 °C in a humidified atmosphere containing 5% CO_2_.

### 2.3. Antibody Staining and Flow Cytometry

Cells were stained with the appropriate antibodies, and fixed by incubation with 1% paraformaldehyde/PBS at 4 °C for more than 1 h. Data were obtained using a BD FACSCalibur flow cytometer (BD Biosciences, San Jose, CA, USA) and analyzed using the FlowJo software, version 10 (BD Biosciences). The following antibodies were used: phycoerythrin (PE)-labeled anti-CD3 (OKT-3, BD Biosciences), APC-labeled anti-PD-1 (EH12.2H7, Biolegend), fluorescein isothiocyanate (FITC)-labeled anti-CD4 (OKT-4, Biolegend), allophycocyanin (APC)-labeled anti-CD8 (SK1, Biolegend), PE-labeled anti-PD-L1 (29E.2A3, Biolegend), FITC-labeled anti-CCR7 (CD197) (REA546; Miltenyi Biotec, North Rhine-Westphalia, Germany), APC-labeled anti-CD45RA (REA1047, Miltenyi Biotec), APC-labeled anti-TIM-3 (F38-2E2, Germany), APC-labeled anti-LAG-3 (CD223) (REA351, Miltenyi Biotec), and APC-labeled anti-TIGIT-APC (REA1004, Miltenyi Biotec). To detect CAR expression, cells were stained with biotin-SP-conjugated AffiniPure goat anti-mouse IgG (Jackson Immuno Research Laboratories, West Grove, PA, USA) and PE-labeled streptavidin (BD Biosciences). The normalized mean fluorescence intensity (MFI) was calculated by dividing the MFI obtained with the sample by the MFI obtained with the isotype-matched control. The percentage of fluorescent cells determined by flow cytometry was verified by manual counting of fluorescent cells in chosen fields under a fluorescence microscope.

### 2.4. Designing and Establishing CRISPR/Cas9 Expression Vectors

**Figure 1:**
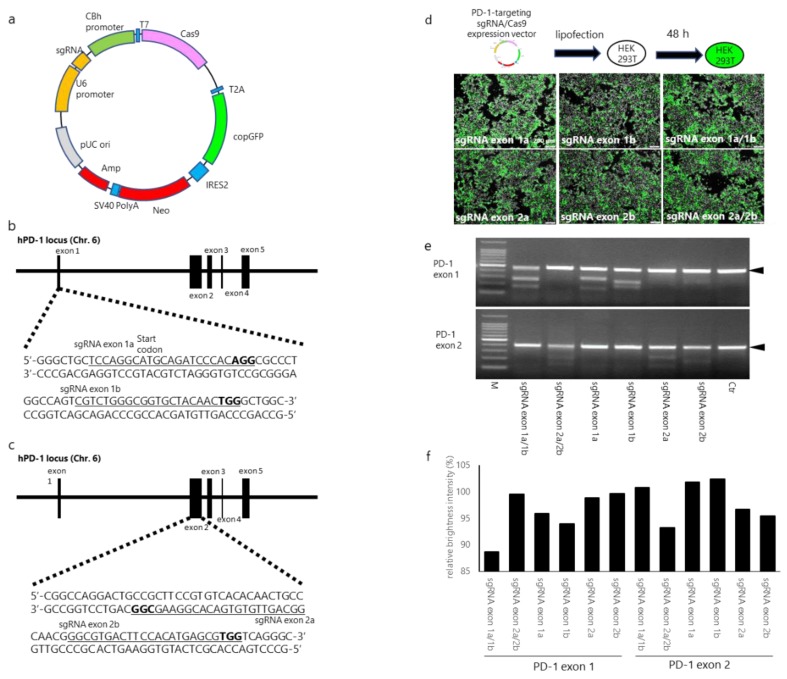
Evaluation of human PD-1- (programmed cell death protein 1)-targeting CRISPR/Cas9 expression vectors in HEK293T cells. (**a**) Schematic representation of the CRISPR/Cas9 expression vector (**b**,**c**). Schematic diagrams of small-guide RNAs (sgRNAs) targeting the PD-1 exon 1 and exon 2 loci. The sgRNA target sites are underlined. Bold letters indicate the proto-spacer adjacent motif (PAM) sequence, which is recognized by Cas9. (**d**) Lipofection of PD-1-targeting CRISPR/Cas9 expression vectors into HEK293T cells. One or two PD-1-targeting sgRNA/Cas9 expression vectors (total, 2 µg DNA) were transfected into HEK293T cells and cultured for 48 h. The images were acquired with a fluorescence inverted microscope. (**e**) PD-1-targeting CRISPR/Cas9 expression vector-mediated DNA cleavage of human PD-1 gene locus (findings from T7EN1-based cleavage assays). The upper and lower panels depict the resolvase-digested PCR product of the PD-1 exon 1 and exon 2 regions, respectively. (**f**) Percent intensity of undigested PCR products of PD-1, as determined by densitometric analysis. Relative brightness intensity (RBI) was calculated by dividing the brightness intensity of each band by the brightness intensity of the control band.

The pUC-U6 promoter sgRNA-CBh promoter-T7-Cas9-T2A from the copepod *Pontellina plumata*-derived green fluorescent protein (copGFP) internal ribosome entry site (IRES)-neomycin (pUC-CRISPR/Cas9/GFP/IRES/Neo) vector was designed based on a pUC-U6-CBh plasmid vector (Figure 1a). sgRNAs were expressed under the control of the U6 promoter. PD-1-targeting sgRNAs were ligated to pUC-CRISPR/Cas9/copGFP/IRES/Neo. The pUC-CRISPR/Cas9/copGFP/IRES/Neo expression vectors were established by GeneCopoeia (Rockville, MD, USA). In the present study, four sgRNAs were designed by visually confirming the protospacer adjacent motif (PAM) sequences of the exon 1 and exon 2 regions of the human PD-1 gene. For PD-1 exon 1, sgRNAs targeting the exon 1a region (5′-TCCAGGCATGCAGATCCCACAGG-3′) and exon 1b region (5′-CGTCTGGGCGGTGCTACAACTGG-3′) were used, and for PD-1 exon 2, previously reported sgRNAs [30] (prepared as positive controls) targeting the exon 2a region (3′-GGCGAAGGCACAGTGTGTTGACGG-5′) and exon 2b region (5′-GGCGTGACTTCCACATGAGCGTGG-3′) were used (Figure 1b,c). Note that the sgRNAs targeting exon 1a included the transcription start sequences (ATG) of PD-1.

### 2.5. Transfection of PD-1-Targeting CRISPR/Cas9 Expression Vectors into HEK293T Cells

The PD-1-targeting CRISPR/Cas9 expression vectors were transfected into HEK293T cells using Fugene 6 (Promega Co., Madison, WI, USA) according to the manual. Briefly, HEK293T cell culture was started the day before transfection so that they had reached 50–80% confluence at the time of transfection. For the transfection, 2 µg each of the CRISPR/Cas9 expression vectors, 6 µL of Fugene 6, and 100 µL of serum-free DMEM were mixed and placed at room temperature for 15 min, and then added to HEK293T cells. After 48 h, the cells were observed with a fluorescence inverted microscope (IX83; Olympus Osaka Japan or ECRIPSE Ti; Nikon, Tokyo, Japan), and transfection efficacy was determined by manually counting the number of copGFP-positive cells.

### 2.6. Construction of SIN Lentiviral Vectors

The EGFRvIII-specific CAR-carrying self-inactivating (SIN) lentiviral vector used here has been previously reported [22]. The 3C10 mAb was originally developed by immunization of mice with a 14-amino acid peptide incorporating an EGFRvIII-specific fusion junction [31], after which its scFv was subsequently cloned [32]. The scFv portion in pELNS-SS1CD28r1BBZeta was replaced with the cDNA for 3C10 scFv by gene synthesis (Genscript, Piscataway, NJ, USA) to establish pELNS-3C10-CAR.

### 2.7. Production of the Vesicular Stomatitis Virus G Glycoprotein Pseudotype of Self-Inactivated Lentivirus

Production of SIN lentiviruses was performed as previously described, with slight modifications [24]. Briefly, HEK293T cells (8 × 10^6^) were cultured in 10% FBS-containing DMEM supplemented with 100 units/mL of penicillin, and 100 μg/mL of streptomycin in T175 flasks (Corning, Corning, NY, USA) for 24 h under standard culture conditions in a humidified atmosphere containing 5% CO2. The cells were co-transfected with the EGFRvIII-specific CAR-carrying SIN lentiviral vector together with the packaging vectors pMDLg/pRRE, pRSV-Rev, and pMD2.G (which were kindly provided by Atsushi Natsume of the Nagoya University School of Medicine), using the Fugene 6, and then incubated for 48 h. The lentivirus-containing culture supernatant was harvested and filtered through a 0.45-μm filter unit (Millipore, Billerica, MA, USA) to remove cellular debris. The filtrated supernatant was mixed with PEG-it Virus Precipitation Solution (5×) (System Biosciences, Palo Alto, CA, USA) and incubated for 24–72 h at 4 °C. This mixture was then centrifuged at 1500× *g* for 30 min at 4 °C, and the pellet was resuspended in the cold sterile medium or PBS (that was 1/20th to 1/10th the volume of the original solution) at 4 °C and stored at −80 °C.

### 2.8. Induction of PD-1-Disrupted Primary Human EvCAR-T Cells

PBMCs were prepared from heparinized peripheral blood obtained from a healthy volunteer using a conventional preparation kit (Lymphoprep; Axis-Shield PoC AS, Oslo, Norway). The PBMCs were transfected with 5 µg of the CRISPR/Cas9 expression vectors or 2.5 µg of the control pmaxGFP vector by Nucleofector 2b (Lonza, Köln, Germany), using the Amaxa Human T cell Nucleofector Kit (VPA-1002; Lonza). Electroporation program V024 was used. After electroporation, the cells were resuspended in AIM-V medium (Thermo Fisher Scientific) containing 10% autoplasma, transferred into a 6-well plate (Corning), and incubated for 4 h at 37 °C in a humidified atmosphere containing 5% CO_2_. The cells were washed and suspended in AIM-V medium supplemented with 200 IU/mL interleukin (IL)-2 (Novartis, Basel, Switzerland) and 10% autoplasma, transferred to 24-well plates (Corning) coated with 5 µg/mL of purified anti-CD3 antibody (OKT-3; Miltenyi Biotec) and 2.5 µg/mL of purified anti-CD28 antibody (15E8; Miltenyi Biotec), and cultured for 24 h under standard culture conditions. The transfection efficiency was determined with a BD FACSCalibur flow cytometer or by manually counting the number of GFP-positive cells. Then, the EvCAR-carrying SIN lentivirus (MOI: 1) was added and centrifuged at 2600 rpm for 45 min at room temperature. After virus infection, the cells were cultured and expanded in AIM-V medium containing 200 IU/mL of IL-2 without autoplasma for 21 days.

### 2.9. Gene Disruption Efficacy of the CRISPR/Cas9 Expression Vectors

Gene-disrupted cells were harvested, and their genomic DNA was extracted using the QIA amp DNA mini kit (Qiagen, Hilden, Germany). The T7 endonuclease-based assay was performed using the Guide-it Mutation Detection Kit (TAKARA Bio, Shiga, Japan) according to the manufacturer’s instructions. Briefly, the targeted regions of PD-1 were amplified from genomic DNA using KOD FX (TOYOBO, Osaka, Japan). The PCR conditions were as follows: 1 cycle at 94 °C for 2 min followed by 40 cycles at 98 °C for 10 s, 63 °C for 30 s, and 68 °C for 30 s, and finally 1 cycle at 68 °C for 7 min. PCR was performed using the thermal cycler Life ECO (Bioer Technologies Co. Ltd., Hangzhou, China). The sequences of the primers used (from Thermo Fischer Scientific) were as follows: PD-1 exon 1: 5′-AGCACTGCCTCTGTCACTCTCG-3′ (forward) and 5′-AAGCCACACAGCTCAGGGTAAG-3′ (reverse), PD-1 exon 2: 5′-GGACAACGCCACCTTCACCTGC-3′ (forward) and 5′-CTACGACCCTGGAGCTCCTGAT-3′ (reverse). The amplification product of the PD-1 exon 1 primers and the PD-1 exon 2 primers were 471 base pairs (bp) and 476 bp in length, respectively. The PCR products were denatured and re-annealed in New England Biolabs (NEB) buffer by using the thermal cycler LifeECO under the following conditions: 95 °C for 5 min, decrease in temperature by 2 °C every second from 95 °C to 85 °C, decrease in temperature by 0.1 °C per second from 85 °C to 25 °C, and decrease in temperature to 4 °C. Rehybridized PCR products were digested with resolvase for 30 min and separated on a 2% agarose gel for 20 min. DNA was visualized under a UV transilluminator (FAS-IV; NIPPON Genetics, Kyoto, Japan). The band intensities of undigested PCR products were quantified using ImageJ (National Institutes of Health, Bethesda, MD, USA), and RBI was calculated for each undigested product by dividing the band intensity by the intensity of the control band.

To assess the off-target mutagenesis of CRISPR/Cas9 expression vectors, we predicted the off-target sites by using an off-targeting potential checking system (https://sg.idtdna.com/site/order/designtool/index/CRISPR_SEQUENCE) designed by Integrated DNA Technologies Inc. (IDT). The system assesses the on- and off-targeting potential of protospacer designs of guide RNAs. The system uses IDT’s proprietary machine learning algorithm for designing high-quality gRNA sequences with high on-target and low off-target activity. The off-target score is calculated by assessing the score of all genomic sites with a sequence similar to the target sequence.

The position of mismatches between the on-target and an off-targets site, along with the total number of potential off-target sites, are also factored into the off-target score. The PCR primers used for the amplification of the target locus are listed in Table S1. PCR reactions were performed under the conditions described earlier. The T7 endonuclease-based assay was performed using the Alt-R Genome Editing Detection Kit (Integrated DNA Technologies Inc.), according to the instructions of the manual.

### 2.10. DNA Sequencing

The KOD FX-amplified PCR products, which had blunt ends, were ligated with pCR-Blunt II-TOPO of the Zero Blunt TOPO PCR cloning kit (Thermo Fischer Scientific), according to the manufacturer’s instructions. Ligation plasmids were used for transformation of the products, and about 50 colonies were picked up. The plasmids were isolated by NucleoSpin Plasmid (MACHEREY-NAGEL, Düren, Germany) and sequenced using the universal primer M13F. Sanger sequencing was performed by GENEWIZ (South Plainfield, NJ, USA).

### 2.11. Analysis of the Inhibitory effects of PD-1-Disrupted EvCAR-T Cells

The xCELLigence RTCA S16 instrument (ACEA Biosciences, San Diego, CA, USA) was used to determine the inhibitory effects of the modified EvCAR-T cells on GBM cells, according to the manufacturer’s instructions. Briefly, complete medium (100 µL) was added to each well, and background impedance on the plates was measured on the xCELLigence RTCA S16 instrument at 37 °C in a humidified atmosphere containing 5% CO_2_. E-plate 16 (ACEA Biosciences) was placed in xCELLigence RTCA S16, and impedance measurements were recorded every 5 min for 72 h. Cells derived from the two human GBM cell lines, U-251MG and Ev-DKMG (2 × 10^4^/well), were seeded in a plate and used as target cells. After 24 h, the expanded EvCAR-T cells (ratio of target to effector cells = 1:0.3) were added to the plates as effector cells. EvCAR-T cell-mediated tumor cell growth inhibition was monitored in real time. Data were analyzed with the RTCA software, version 1.2 (ACEA Biosciences). Relative growth inhibition was calculated using the following formula: relative normalized CI (%) = 100 − (normalized CI of each sample/normalized CI of T cells) × 100.

### 2.12. Statistical Analyses

Statistical analyses were carried out using Prism 8 (GraphPad Software Inc., San Diego, CA, USA). The results are shown as mean ± standard deviation (SD). The statistical significance of differences was determined using a *t*-test or two-way analysis of variance followed by Sidak multiple comparison. *p* values that were < 0.05 were considered to indicate statistical significance.

## 3. Results

### 3.1. sgRNA/Cas9-Induced Disruption of Human PD-1

The CRISPR/Cas9 expression vectors that were designed in this study are shown in Figure 1a: two human PD-1 exon 1-targeting small-guide RNAs (sgRNAs) and two PD-1 exon 2-targeting sgRNAs were designed and ligated into the CRISPR/Cas9 expression vector. The sgRNA sequences and their target regions in the PD-1 gene sequence are shown in Figure 1b and c. The sgRNA target sequences for PD-1 exon 2 were previously reported [30], and these sequences were used as positive controls (Figure 1c).

The simultaneous use of two sgRNAs for different target sites has been shown to significantly improve the gene editing efficiency of the CRISPR/Cas9 system in a mouse model [33]. To confirm this, a single PD-1-targeting CRISPR/Cas9 expression vector (sgRNA exon 1a, 1b, 2a, and 2b), or a combination of PD-1 exon 1-targeting CRISPR/Cas9 expression vectors (sgRNA exon 1a/1b) or PD-1 exon 2-targeting CRISPR/Cas9 expression vectors (sgRNA exon 2a/2b) was transfected into HEK293T cells. The copGFP was examined at 48 h after electroporation by fluorescence microscopy: 40–50% of the cells were positive for copGFP in all the transfection settings (Figure 1d). Mutation detection analysis revealed that the cleavage bands were all observed in the cells transfected with the CRISPR/Cas9 expression vectors, but they were not observed in non-transfected cells (Figure 1e). Densitometric analysis showed that the relative brightness intensity (RBI) of sgRNA exon 1a, sgRNA exon 1b, and sgRNA exon 1a/b was 95.9%, 93.9%, and 88.7%, respectively, in the PD-1 exon 1 region, and the RBI of sgRNA exon 2a, sgRNA exon 2b, and sgRNA exon 2a/b was 96.6%, 95.4%, and 93.2%, respectively, in the PD-1 exon 2 region (Figure 1f). The transfection of all PD-1-targeting CRISPR/Cas9 expression vectors in HEK293T cells resulted in a decrease in the RBI of the PCR products. The combination of sgRNA exon 1a/1b- and sgRNA exon 2a/2b-targeting vectors resulted in a lower RBI than a single PD-1-targeting CRISPR/Cas9 expression vector. Further, the combined sgRNA exon 1a/1b vectors resulted in a lower RBI than the combined sgRNA exon 2a/2b vectors. The results indicate that the combined sgRNA exon 1a/1b vectors efficiently disrupted the PD-1 gene. This combination of vectors was therefore used for the following experiments.

### 3.2. Establishment of PD-1-Disrupted EvCAR-T Cells using PD-1 Exon 1-Targeting CRISPR/Cas9 Expression Vectors

**Figure 2:**
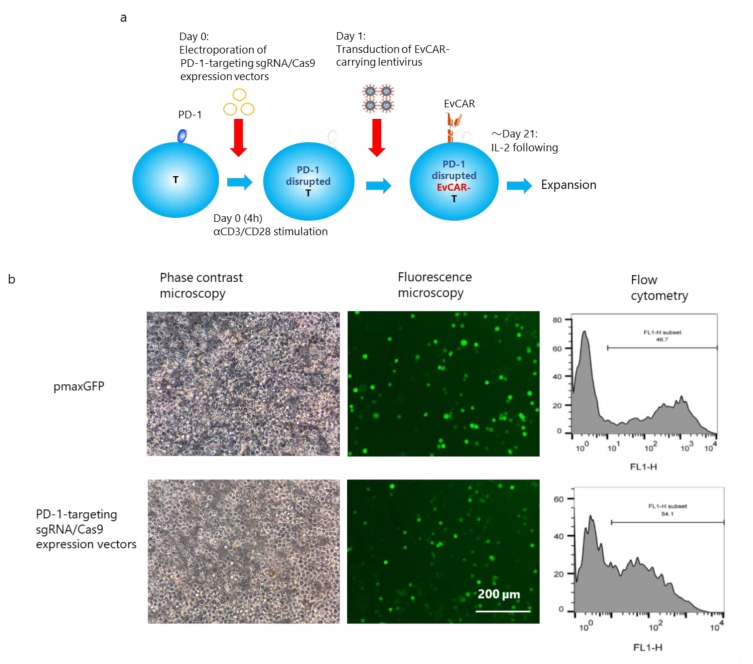

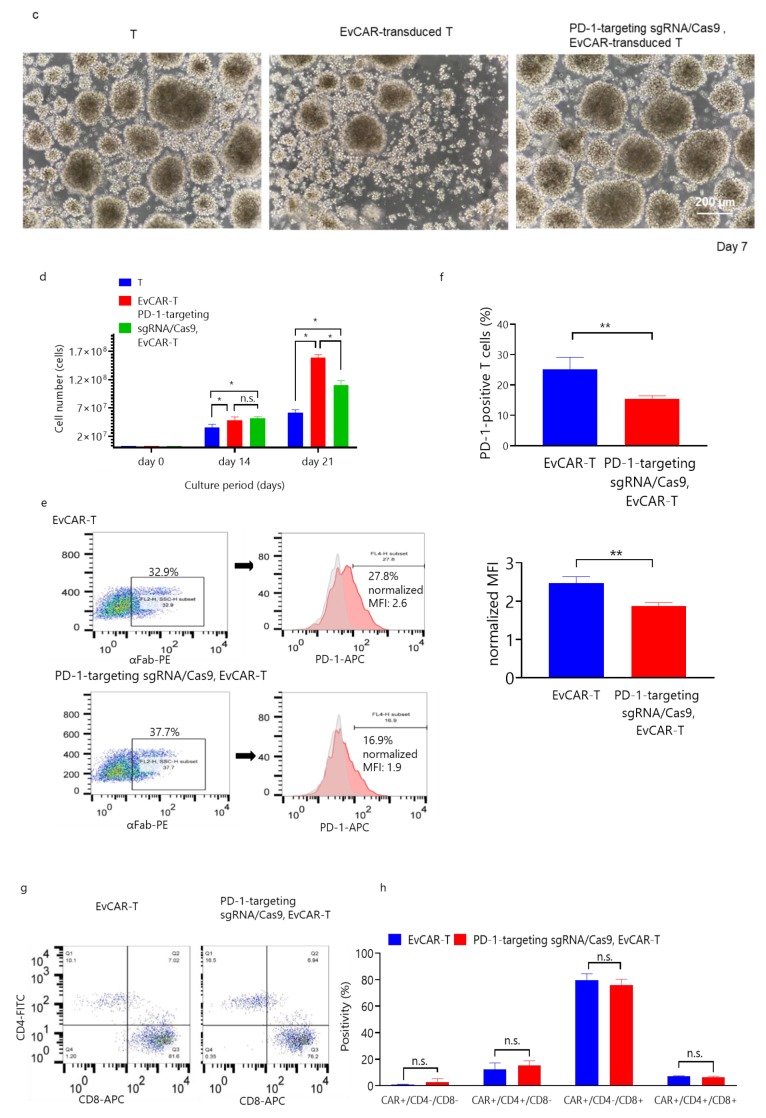
PD-1 gene disruption by sgRNA/Cas9 expression vectors in EGFRvIII-targeting chimeric antigen receptor (EvCAR-T) cells derived from human peripheral T cells. (**a**) Schematic representation of the procedure for introducing PD-1 sgRNA/Cas9 expression vectors and EvCAR into human T cells. (**b**) Transfection efficacy of PD-1-targeting sgRNA/Cas9 expression vectors. The upper and lower panels depict the electroporation of control pmaxGFP vectors and PD-1-targeting sgRNA/Cas9 expression vectors in human T cells, respectively. The images were acquired under a phase contrast microscope and fluorescence microscope (left, middle), and flow cytometric data are shown on the right. (**c**) T cells at day 7 after transfection, as observed under an inverted microscope. The left, middle, and right photos show T cells, EvCAR-transduced T cells, and PD-1-targeting sgRNA/Cas9 vector- and EvCAR-transduced T cells, respectively. (**d**) Control T cells, EvCAR-transduced T cells populations and PD-1-targeting sgRNA/Cas9 vector- and EvCAR-transduced T cells populations were cultured in vitro upon stimulation with exogenous IL-2 for 21 days. The total cell number was counted on day 14 and day 21. Data show the mean ± standard deviation (SD) for four experiments. The significance of differences was determined by two-way ANOVA followed by Tukey’s test. *p < 0.05. (**e**) Representative results for the expression of PD-1 on αFab (EvCAR)-expressing T cells, as determined by flow cytometry at day 21 after electroporation. The upper and lower panels depict EvCAR-transduced T cells and PD-1-targeting sgRNA/Cas9 expression vector-transduced T cells, respectively. The left and right panels show EvCAR and PD-1 expression data, respectively. PD-1 expression was determined by gating EvCAR-positive cells. (**f**) Percentage of PD-1 positive cells (left) and normalized mean fluorescent intensity (MFI) (right) for PD-1 expression are shown. Data show the mean ± standard deviation (SD) for four experiments. The significance of differences was determined by the t-test. **p < 0.05. (**g**) Representative data for the frequency of CD4- and CD8-positive cells among EvCAR (left) and PD-1-targeting sgRNA/Cas9 vector- and EvCAR-transduced T cell populations (right). (**h**) Frequency of CD4/CD8 double-positive, CD4/CD8 double-negative, CD4 single-positive, and CD8 single-positive cells among EvCAR and PD-1-targeting sgRNA/Cas9- and EvCAR-transduced T cells populations. Data show the mean ± standard deviation (SD) for four experiments. Significance was determined with the t-test.

The experimental protocol for induction of PD-1-disrupted EvCAR-T cells is shown in Figure 2a. Peripheral blood mononuclear cells (PBMCs) collected from a healthy volunteer were transfected with the pmaxGFP vector or the sgRNA exon 1a/1b-targeting CRISPR/Cas9 expression vectors through electroporation. After the transfection, flow cytometric analysis showed that 50% of the cells transfected with the pmaxGFP vector and sgRNA exon 1a/1b-targeting CRISPR/Cas9 expression vectors were positive for copGFP (Figure 2b). Next, EvCAR-carrying lentivirus was added, and the cells were allowed to undergo expansion for 21 days following IL-2 stimulation, following which the in vitro growth was assessed based on the total cell number. T-cell clones were observed on day 7, after which they continued to expand over the course of the following days; this is indicative of good proliferation and activation of the T cells (Figure 2c). On day 14, the number of CRISPR/Cas9 expression vector- or pmaxGFP vector-, EvCAR-carrying lentivirus-transduced T cells was higher than the number of control T cells (*p* < 0.05), and transfection of the CRISPR/Cas9 expression vectors did not affect EvCAR-carrying lentivirus-infected T-cell growth (*p* > 0.05). On day 21, the number of EvCAR-carrying lentivirus-infected T cells transfected with the CRISPR/Cas9 expression vectors or pmaxGFP vector was higher than the number of control pmaxGFP vector-transfected T cells (*p* < 0.05), but the transfection of CRISPR/Cas9 expression vectors inhibited the growth of EvCAR-carrying lentivirus-infected T cells (*p* < 0.05) (Figure 2d). Thus, transfection of the CRISPR/Cas9 vectors had a slightly inhibitory effect on T-cell growth.

On day 21, the expression of PD-1 in EvCAR-T cells was examined. Among the EvCAR-T cell population transfected with the pmaxGFP and CRISPR/Cas9 expression vectors, 29.5 ± 2.0% and 38.4 ± 3.8% (mean ± SD%) were positive for EvCAR, respectively. Thus, a significantly higher number of EvCAR-T cells transfected with the CRISPR/Cas9 expression vectors than non-transfected EvCAR-T cells were positive for EvCAR (data not shown). With regard to PD-1 expression, 25.1 ± 4.0% and 15.5 ± 1.0% of EvCAR-positive T cells and the CRISPR/Cas9 expression vector-transfected EvCAR-T cells were positive for PD-1, respectively (Figure 2e,f). A 38.3% reduction of PD-1-positive cells was observed in the CRISPR/Cas9 expression vector-transfected EvCAR-T cells, as compared with the EvCAR-T cells. The MFI of PD-1 was 2.48 ± 0.16 and 1.87 ± 0.09 in non-transfected EvCAR-T cells and CRISPR/Cas9 expression vector-transfected EvCAR-T cells, respectively (Figure 2e,f). A 24.5% reduction in PD-1 expression was observed in the CRISPR/Cas9 expression vector-transfected EvCAR-T cells in comparison with non-transfected EvCAR-T cells. Thus, the level and expression of PD-1 in EvCAR-T cells were significantly inhibited by the CRISPR/Cas9 expression vectors (*p* < 0.01 for PD-1 positivity, *p* < 0.01 for normalized MFI). Population analysis of CD4- and CD8-expressing cells showed that the CRISPR/Cas9 expression vectors did not affect the number of CD4/CD8 double-positive, CD4/CD8 double-negative, CD4 single-positive, and CD8 single-positive EvCAR-T cells (Figure 2g,h). Further analysis revealed that CRISPR/Cas9 expression vectors also did not affect the naïve/effector/memory phenotype and expression of the checkpoint molecules T-cell immunoglobulin and mucin domain-3 (TIM-3), lymphocyte activation gene-3 (LAG-3), and T-cell immunoreceptor with immunoglobulin and immunoreceptor tyrosine-based inhibitory motif (ITIM) domains (TIGIT) on EvCAR-T cells (Figure S1a,b).

### 3.3. Disruption of the PD-1 Gene in Human Primary EvCAR-T Cells by PD-1-Targeting CRISPR/Cas9 Expression Vectors

**Figure 3:**
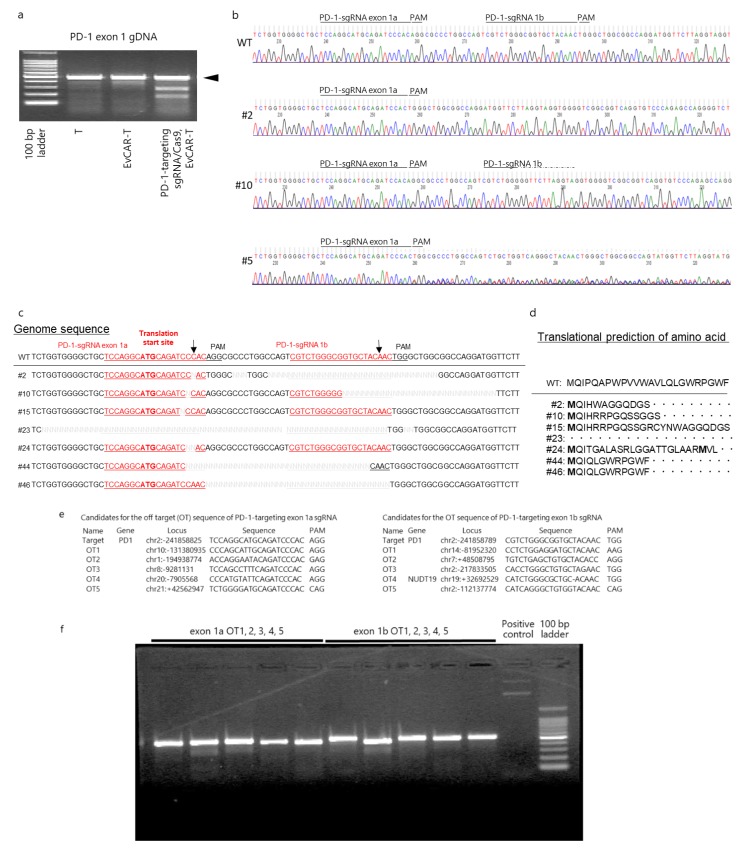
Genomic PD-1 exon 1 disruption in EvCAR-T cells transduced with the PD-1-targeting sgRNA/Cas9 vectors (**a**) PCR products were amplified and subjected to mutation detection assay. The electrophoresis bands represent the 100-bp ladder, control T cell populations, EvCAR-transduced T cell population, and PD-1-targeting sgRNA/Cas9-, EvCAR-transduced T cell population (from left to right). (**b**) DNA sequences of PD-1-targeting sgRNA/Cas9 expression vectors- and EvCAR-transduced T cell population, as determined by Sanger sequencing analysis. Representative sequence data are depicted. WT indicates wild type; # indicates clone numbers. (**c**) The panel shows the DNA sequence data for exon 1a and exon 1b. (**d**) The panel shows the predicted amino acid sequence after translation. Off-target cleavage assessment by T7E1 assays. (**e**) Candidates for the CRISPR/Cas9 expression vectors’ off-target (OT) sequences. The left and right tables show the OT sequences of PD-1-targeting exon 1a and 1b sgRNA, respectively. (**f**) The photo depicts the predicted off-target locus PCR product digested by T7E1 of PD-1-disrupted EvCAR-T cells.

To examine in detail the PD-1 gene disruption effect of the PD-1-targeting CRISPR/Cas9 expression vectors, mutation detection assay was performed on the expanded cells on day 21. The assay showed that the PD-1 gene in the genome was clearly cleaved by the CRISPR/Cas9 expression vectors (Figure 3a). Further, clonal DNA sequencing showed that 13 out of a total of 50 (26.0%) clones had different sequences around the target sites. The representative Sanger sequencing chromatograms of DNA derived from PD-1-disrupted EvCAR-T cells are shown in Figure 3b: Three clones, including clone #2, had deletions around the sgRNA exon 1a-argeting site. Four clones, including clone #10, had deletions around the sgRNA exon 2a-targeting site. The genome sequences corresponding to the deletions of these 7 clones are shown in Figure 3c. In addition, the DNA sequence of the sgRNA target site could not be read after sgRNA exon 1a for 6 clones, including clone #5 (Figure 3b). Among the observed indels, the deletion mutants were the most prominent. Amino acid translation prediction of the deletion clones demonstrated that the shift of the genomic reading frame occurred downstream of the sgRNA exon 1a targeting site. Additionally, clone #23 did not have a translation start codon, and clone #24 had two translation start codons in the displayed region (Figure 3d).

In addition, assessment of the off-target effects of the CRISPR/Cas9 expression vectors on genomic DNA revealed that they did not result in distinct insertions or deletions in the off-target sites predicted by the off-targeting potential detection system (Integrated DNA Technologies Inc., Coralville, IA USA) (Figure 3e,f).

### 3.4. Expression of PD-1 in PD-1-Disrupted EvCAR-T Cells and their Effects on the Growth of GBM Cells

**Figure 4:**
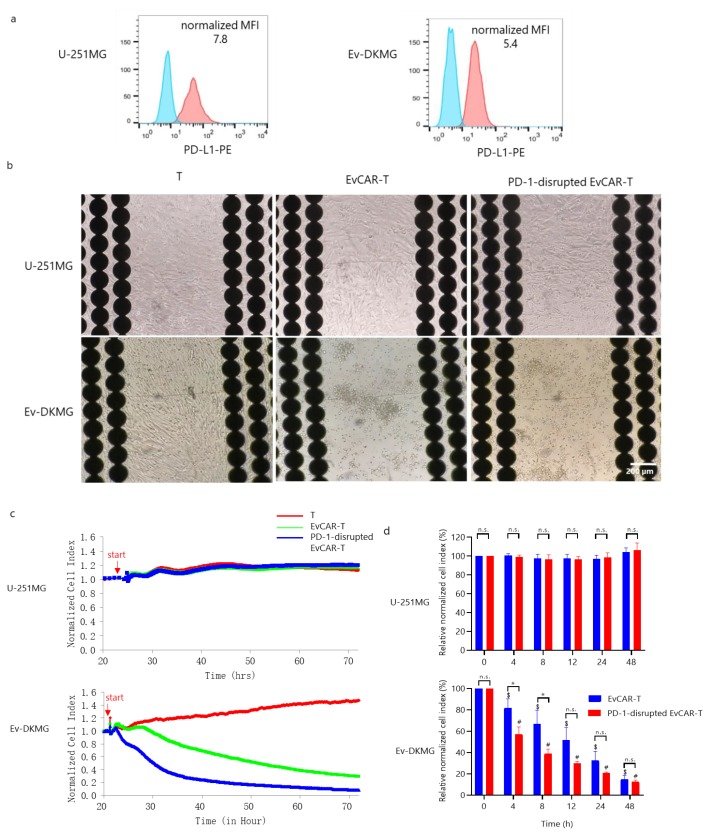
Enhanced growth inhibition of human glioblastoma (GBM) cells by CRISPR/Cas9-mediated PD-1 disruption in EvCAR-T cells. (**a**) PD-L1 expression in U-251MG and EGFRvIII-expressing DKMG cells. The upper and lower panels depict U-251MG and EGFRvIII-expressing DK-MG GBM cells, respectively. The blue and red curves represent the isotype control and anti-PD-L1 antibody, respectively. (**b**) Images of GBM cell lines co-cultured with T cells, EvCAR-T cells, and PD-1-disrupted EvCAR-T cells (left, middle, and right, respectively) at 72 h after culture. The upper panels and lower panels depict U-251MG and EGFRvIII-expressing DKMG cells, respectively. (**c**) Representative graphs of the RTCA-based growth inhibition assay. U-251MG (upper graph) and EGFRvIII-expressing DKMG (Ev-DKMG, lower graph) cells were co-cultured with T cells (red line), EvCAR-T cells (green line), or PD-1-disrupted EvCAR-T cells (blue line). The X and Y axis represent the co-culture time and normalized cell index respectively. The red arrow indicates the start of culture. (**d**) Graphs depicting RTCA-based growth inhibition assays. The upper and lower graphs depict the data for U-251MG and Ev-DKMG cells, respectively. The X and Y axes represent co-culture time and relative normalized cell index. Statistical differences were determined by two-way ANOVA. *^,#,$^ *p* < 0.05.

First, the expression of PD-L1 in two GBM cell lines, U-251MG and EGFRvIII-expressing DKMG (abbreviated Ev-DKMG), was evaluated. The two cell lines exhibited strong expression of PD-L1, with normalized MFI values of 7.8 and 5.4, respectively (Figure 4a). These values indicate that the two cell lines were suitable for testing the anti-tumor ability of EvCAR-T cells. Therefore, the growth inhibitory effects of EvCAR-T cells on the two GBM cell lines were determined using the real-time cell analysis (RTCA) system. It has been previously reported that the RTCA system utilizes cellular impedance readouts to monitor real-time changes in cell number, cell size, and cell-substrate attachment strength, which are together represented by a parameter called the cell index (CI) that reflects the cell number of target cancer cells. Importantly, the RTCA data have been found to be comparable with the data from cytotoxicity assays including the Cr^51^-release assay [34,35,36,37]. U-251MG and Ev-DKMG cells were seeded and cultured for 1 day, and then, T cells, EvCAR-T cells, and PD-1-disrupted EvCAR-T cells cultured for 14 days were added to the wells at an effector-to-target ratio of 0.3:1. The number of co-cultured EvCAR-T cells and PD-1-disrupted EvCAR-T cells was adjusted according to the EvCAR expression level. T cells and EvCAR-T cells displayed a negligible impedance signal, as confirmed before the test, since these cells are not adherent by nature. After the assays, each well of the E-plates was photographed under a fluorescence microscope. EvCAR-T cells and PD-1-disrupted EvCAR-T cells had an obvious inhibitory effect on the Ev-DKMG cells, but there was no inhibitory effect on U-251MG cells (Figure 4b). The PD-1-disrupted EvCAR-T cells and EvCAR-T cells caused a rapid decrease in the normalized CI of Ev-DKMG cells, but did not decrease the normalized CI of U-251MG cells, compared to control T cells (Figure 4c). The percentage of normalized CI was calculated by dividing the normalized CI of PD-1-disrupted EvCAR-T cells or EvCAR-T cells by the normalized CI of T cells. Compared to EvCAR-T cells, PD-1-disrupted EvCAR-T cells caused a significant decrease in the percentage of normalized CI of Ev-DKMG cells at 4 and 8 h (*p* < 0.05), but did not cause any significant decrease in the corresponding CI of U-251MG cells (Figure 4d). These results indicate that the PD-1-targeting CRISPR/Cas9 expression vectors enhance the growth inhibitory effects of EvCAR-T cells via the disruption of genomic PD-1.

## 4. Discussion

To the best of our knowledge, this is the first study to report the disruption of the PD-1 gene by two PD1 exon 1-targeting sgRNAs and Cas9 expression vectors in third-generation EvCAR-T cells (under the control of CD3zeta, CD28, and 4-1BB signaling molecules) derived from healthy human T cells. In a similar published study, Choi et al. reported that CRISPR-Cas9 disruption of PD-1 enhances the activity of second-generation EvCAR-T cells under the control of CD3zeta and 4-1BB signaling molecules in a preclinical model of human GBM [38]. However, our study is distinguished by the use of PD-1-disrupted third-generation EvCAR-T cells, as opposed to the second-generation EvCAR-T cells used in the Choi et al. study [38].

In the present study, PD-1 disruption was found to enhance the growth inhibitory effect of EvCAR-T cells on GBM cells. We have explored the disruption of PD-1 by CRISPR/Cas9 genome editing technologies and have reported a non-viral-mediated electroporation technique that may be suitable for clinical application. This method was found to be feasible and efficient for PD-1 gene disruption by electroporation of double plasmids encoding double sgRNAs and Cas9. This method enhanced the inhibitory effect of EvCAR-T cells on GBM cell growth. Additionally, the sgRNAs targeting PD-1 exon 1 that were designed in this study slightly inhibited EvCAR-T cell expansion. Therefore, our sgRNAs appear to have some off-target effect on T-cell growth-related genes. Although the possibility of inducing p53-mediated DNA damage by genome editing with CRISPR/Cas9 has been discussed [39], we believe that this effect of the sgRNA on the growth of T cells does not pose a risk of cancer occurrence or progression. In this study, we also showed that EvCAR-T cells and PD-1-disrupted CAR-T cells did not inhibit non-EGFRvIII-expressing U-251MG cells. This result was consistent with the findings reported for PD-1-disrupted mesothelin-targeted CAR T cells [40]. PD-1 inhibits CD3/28 signaling via PI3K [41], but EvCAR-T cells do not recognize non-EGFRvIII-expressing cells and do not activate PI3K via CD3zeta/CD28. Therefore, the disruption of the PD-1 gene is not likely to affect non-target cells under the present experimental conditions. The present results highlight the therapeutic ability of EvCAR-T cells disrupted by the sgRNA/Cas9 expression vectors designed here, and lay the basis for EvCAR-T cell-based immunotherapy against GBM.

We designed sgRNAs against exon 1a (5′-TCCAGGCATGCAGATCCCACAGG-3′) and 1b (5′-CGTCTGGGCGGTGCTACAACTGG-3′). Exon 1a included a translation start codon and has not been previously reported. Importantly, this combination of sgRNAs against PD-1 exon 1 has not been reported so far, and it could have important implications for studies on the expression of PD-1. With regard to some of the other studies that have reported sgRNAs against PD-1, Ren et al. disrupted PD-1 with a single PD-1 exon 1-targeting sgRNA (5′-GGCCAGGATGGTTCTTAGGT-3′) in second-generation CD19 or prostate stem cell antigen-redirected CAR-T cells under the control of the CD28 molecule as the co-stimulatory domain and CD3ζ [42]. Rupp et al. also reported disruption of the PD-1 gene by an exon 1-targeting sgRNA (5′-CGACTGGCCAGGGCGCCTGT-3′) in second-generation CD19-redirected CAR-T cells [43]. In addition, Guo et al. also reported the transcripts of dual PD-1 exon 1-targeting sgRNAs (5′-GTCTGGGCGGTGCTACAACT-3′ and 5′-GGCCAGGATGGTTCTTAGGT-3′) that were used to disrupt the PD-1 gene in second-generation glypican-3-targeting CAR-T cells [44]. The exon 1b sgRNA (5′-CGTCTG GGCGGTGCTACAACTGG-3′) designed in the present study targeted a similar area as the sgRNA designed by Guo et al. (the sgRNA sequence designed by Guo et al. is underlined). Another such study was the one by Hu et al., which reported the disruption of PD-1 by a transcript of a single PD-1 exon 1-targeting sgRNA (5′-CCCACAGGCGCCCTGGCCAGTCG-3′) in second-generation mesothelin-redirected CAR-T cells [40]. Finally, Su et al. also reported the PD-1 disruption effect of two PD-1 exon 2-targeting sgRNAs (5′-GTGACTTCCACATGAGCGTGG-3′ and 5′-CCGCTTCCGTGTCACACAACTGCC-3′) in cancer-antigen-specific T cells derived from cancer patients [30].

RTCA analysis revealed that PD-1 disruption mediated by our sgRNA/Cas9 expression vectors enhanced the effect of the EvCAR-T cells under the control of CD28z, 4-1BB, and CD3zeta as signaling domains on Ev-DKMG. PD-1-disrupted EvCAR-T cells had a more significant inhibitory effect on Ev-DKMG cell growth than EvCAR-T cells at 4 and 8 h (Figure 4d).

In the current study, the PD-1-targeting sgRNA/Cas9 expression vectors resulted in 9.4% reduction of PD-1 expression in EvCAR-T cells. These PD-1-disrupted EvCAR-T cells including population comprised 38.4% EvCAR-positive cells, and PD-1 gene disruption was assumed to 24.4% cells in EvCAR-transduced cellular populations. Sanger DNA sequencing analysis revealed that the sgRNA/Cas9 expression vectors disrupted the PD-1 gene in 26.0% of the clones. Based on these findings, the PD-1 gene disruption level corresponded to the PD-1 expression findings. We hypothesized the possibility that the sequence-dependent cloning efficiency of the PCR product make a discrepancy between the results of the expression of PD-1 and genomic PD-1 disruption level. However, these data almost showed no gap in these findings.

RTCA analysis showed that PD-1 disruption enhanced EvCAR-T cell-mediated growth suppression of Ev-DKMG. These results demonstrate that the sgRNA/Cas9-mediated antitumor effects of EvCAR-T cells are strongly dependent on PD-1 disruption (Figure 4d). We have confirmed the T-cell phenotype; the expression of the immune checkpoint receptors LAG-3, TIM-3, and TIGIT; and off-target effect on the genome in PD-1-disrupted EvCAR-T cells. These results indicate that distinct changes were not observed in the PD-1-disrupted EvCAR-T cells transduced with the CRISPR/Cas9 expression vectors. The data strongly imply that even a 10% disruption is enough to inhibit PD-1 function. In addition, it should be considered that the RTCA-based cytotoxicity assay is as sensitive as the standard cytotoxic assays. Therefore, we consider that the knockdown efficiency was sufficient for this study. However, further analysis (e.g., analysis of off-target sites) of single cell level, using next-generation sequencing, is required to confirm the specificity of the designed vectors for PD-1.

Several clinical trials have examined the effects of CAR-T cells on GBM. In one such study, O’Rourke et al. reported that a single intravenous infusion of EvCAR-T cells resulted in on-target activity in the brain. This study revealed that CAR-T cells overcome the blood–brain barrier to reach the GBM tumor [45]. In another study, Brown et al. reported that IL-13 receptor alpha 2-redirected CAR-T cell therapy resulted in the regression of GBM [46,47]. Further, Ahmed et al. reported that HER2-redirected CAR T cells specific for the CMV.pp65 antigen reduced tumor volume and was clinically beneficial in 33% of GBM patients [48], and Shiina et al. reported that podoplanin-targeting CAR-T cells inhibited orthotopic GBM in a mouse brain model [49]. Based on these findings, the PD-1-disrupted EvCAR-T cells in the present study, which exhibited antitumor effects in the GBM cell line, should be investigated for their effects on GBM in the clinical setting.

The present study has some limitations. One of the main limitations is that we evaluated the antitumor effect of PD-1-disrupted EvCAR-T cells against GBM only in in vitro conditions. In the future, we need to examine the in vivo anti-tumor effect of PD-1-disrupted EvCAR-T cells on xenografted GBM in highly immuno-incompetent mice, such as NOG (non-obese diabetes/severe combined immune-deficient/IL-2 receptor gamma null) mice. Another limitation is the use of adherent cell lines, as they may not accurately simulate glioma progression in mice. Typically, xenografts simply grow by pushing against brain tissue without ever interacting with it. Therefore, 3D glioma neurospheres or patient organoids might be more useful models than serum-grown adherent cell lines as a basis for future preclinical trials. Further, we used blood from healthy volunteers in this study, but expanding lymphocytes induced from the blood of patients with cancers is challenging because of the possibility of immune function disorders [50]. This limitation might also be true for ex vivo T-cell amplification. For example, the decreased expression of CD3ζ chains in cancer patients is not reversed by IL-2 administration [51]. Finally, in the present study, the percentage of edited CAR-T cells obtained with the CRISPR/Cas9 technique is fairly low. A high number of PD-1-disrupted EvCAR-T cells would be required to induce a detectable effect on in vivo tumor growth. The inhibition of PD-1 expression could be increased by directly incorporating the Cas9 protein and sgRNA into T cells. This possibility needs to be explored before preclinical trials are designed.

## 5. Conclusions

Our sgRNA/Cas9 expression vectors precisely disrupted the target region of PD-1 and inhibited the expression of PD-1 in EvCAR-T cells. The PD-1-disrupted EvCAR-T cells had an in vitro growth inhibitory effect on EGFRvIII-expressing GBM cells without altering the T-cell phenotype and the expression of other checkpoint receptors. In the future, this vector must be studied for its in vivo effects in order to determine its potential as a clinically applicable tool for inducing PD-1-disrupted EvCAR-T cells, and to improve the efficacy of EvCAR-T cell-based adoptive immunotherapy for GBM.

## Supplementary Materials

The following are available online at https://www.mdpi.com/2073-4409/9/4/998/s1, Figure S1: Effect of the CRISPR/Cas9 expression vectors on the phenotype of EvCAR-T cells, Figure S2: EGFR, PD-1, and PD-L1 expression in gliomas, Table S1: Sequences of the PCR primers used for amplification of the predicted off-target locus.s1.
