# Supplementary material for: Effect of CRISPR/Cas9-Mediated PD-1-Disrupted Primary Human Third-Generation CAR-T Cells Targeting EGFRvIII on In Vitro Human Glioblastoma Cell Growth
Source: Cells. 2020 Apr 16;9(4):998. doi: 10.3390/cells9040998 (PMC7227242; doi:10.3390/cells9040998)
Supplement: Supplementary file 1 [file cells-09-00998-s001.pdf]

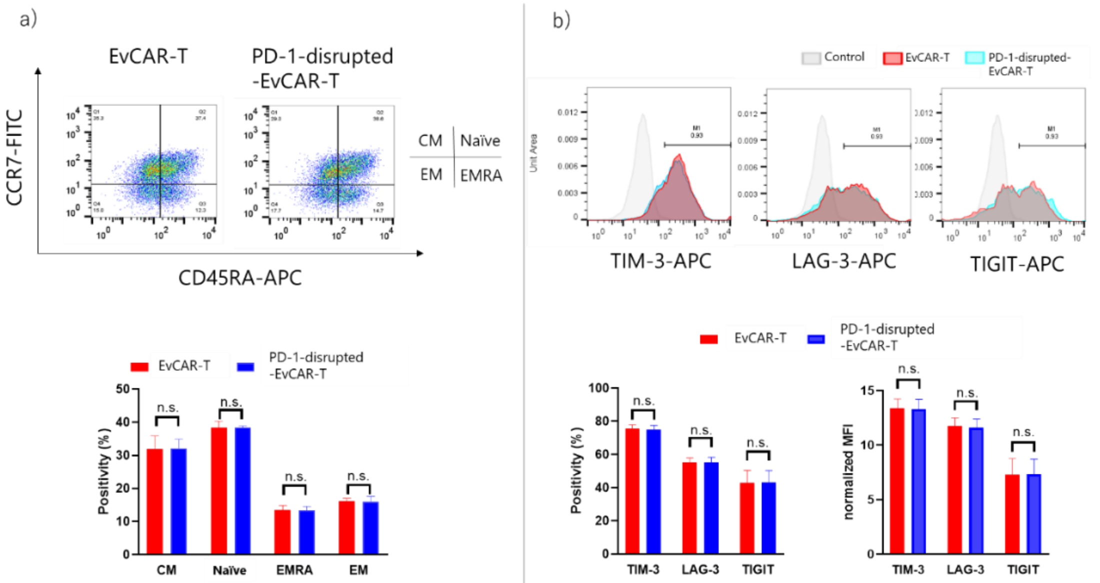

d

Figure S1. Effect of the CRISPR/Cas9 expression vectors on the phenotype of EvCAR-T cells. (a) The upper panels depict representative flow cytometric data for CCR7 and CD45RA expression in CAR-positive cells. The left and right panels show EvCAR-T cells and PD-1-disrupted EvCAR-T cells, respectively. CD45RA+/CCR7+, CD45RA+/CCR7-, CD45RA-/CCR7+, and CD45RA-/CCR7- cells represent naïve, effector memory CD45RA-positive subset (EMRA), central memory (CM), and effector memory (EM) T cells, respectively. The lower graphs depict the positivity of the indicated cell populations for the tested receptors. Data show the mean ± standard deviation (SD) values for four experiments. Significance was determined with the *t*-test. n.s. indicates not significant. (b) The upper panels depict TIM-3, LAG-3, and TIGIT expression on CAR-positive cells. The red, blue, and gray histogram plots show EvCAR-T, PD-1-disrupted EvCAR-T, and control cells, respectively. M1 is set to less than 1% of the control cells. The lower graph depicts positivity (left) and normalized mean fluorescent intensity (MFI) (right) of EvCAR-positive cells. The red and blue bars indicate EvCAR-T cells and PD-1-disrupted EvCAR-T cells, respectively. The data for normalized MFI are calculated as follows: individual MFI was divided by the control MFI value. Data show the mean ± standard deviation (SD) values for four experiments. Significance was determined with the *t*-test. n.s. indicates not significant.

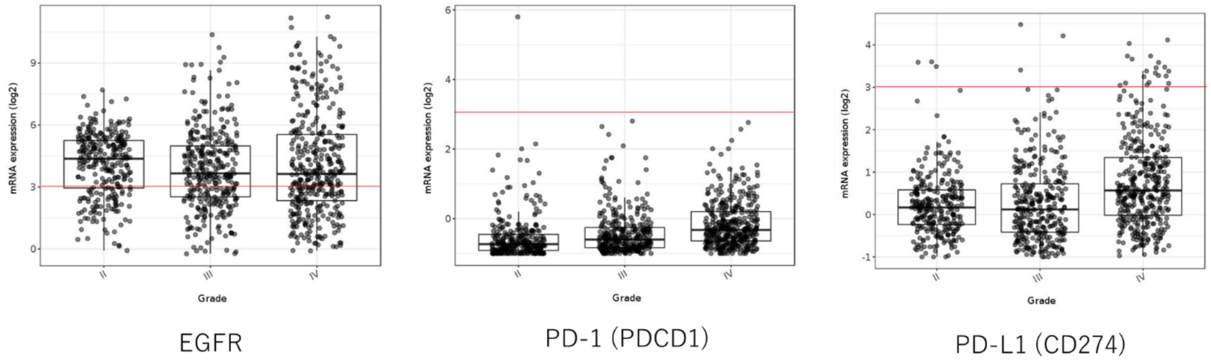

Figure S2. EGFR, PD-1, and PD-L1 expression in gliomas. The X-axis and Y-axis depict mRNA expression and the glioma grade according to WHO classification, respectively. When the log2-transformed mRNA expression is less than 3, expression is considered as negative (red line). The data shown are reproduced from the GlioVis database (http://gliovis.bioinfo.cnio.es/).

Primer name	Sequence(5'-3')	length (bp)
PD-1-exon 1a-OT1 F	GTAACCACAGGTGACAGAGAAA	506
PD-1-exon 1a-OT1 R	CTGTGGCTGTTCTGGAGATT	
PD-1-exon 1a-OT2 F	AAGCCACCATTTGCCATAAAC	512
PD-1-exon 1a-OT2 R	CTGTGCCCTTCAGAAGTATCTC	
PD-1-exon 1a-OT3 F	TGCTTTGGTCATCCACCATTA	513
PD-1-exon 1a-OT3 R	GCTACAGATACTGCTTCTCACTC	
PD-1-exon 1a-OT4 F	TGCTGCACTGGGCTTAAT	497
PD-1-exon 1a-OT4 R	CTCCAGTACAAATGGCTAGGAC	
PD-1-exon 1a-OT5 F	CTGACCTGGAAATGGCTTCT	477
PD-1-exon 1a-OT5 R	TTCCCTCCCAGGGTATTCA	
PD-1-exon 1b-OT1 F	CAATGCCTGCCAAGAAATGAA	510
PD-1-exon 1b-OT1 R	GATTTGGGCTTGAGGGAGAA	
PD-1-exon 1b-OT2 F	CCTGTGCCTCATTGCCTAATA	462
PD-1-exon 1b-OT2 R	GCTAAATTCTAAGCCAGCTCAAAG	
PD-1-exon 1b-OT3 F	AAACCAATGGCGGTTGAATG	515
PD-1-exon 1b-OT3 R	GTAAATGGCCAGGGAAAGGA	
PD-1-exon 1b-OT4 F	ACGTAGCCTTCCGCATCT	500
PD-1-exon 1b-OT4 R	AGAGTTTCCAGCCCGTCTAA	
PD-1-exon 1b-OT5 F	CCTCGCCAAACACCTAATCT	518
PD-1-exon 1b-OT5 R	TGCTCAAGGGAAGGAGAAAC	

Table S1. Sequences of the PCR primers used for amplification of the predicted off-target locus
